# Coping with Job Resources for Female Employees’ Thriving at Work: A Mediated Moderation Model of Benevolent Sexism and Self-Efficacy

**DOI:** 10.3390/bs15050640

**Published:** 2025-05-08

**Authors:** Po-Chien Chang, Shuyue Liu, Yuanli Guo, Qihai Cai

**Affiliations:** 1 School of Business, Macau University of Science and Technology, Macau SAR 999078, China; 2 Office of Academic Affairs, Guizhou University of Finance and Economics, Guiyang 550025, China

**Keywords:** benevolent sexism, self-efficacy, job resources, thriving at work, work–home resources model

## Abstract

Building on the work–home resources model, this study develops a mediated moderation model to examine how benevolent sexism within intimate relationships influences female employees’ thriving at work through job resources and self-efficacy. In this cross-sectional study, we surveyed 209 married female employees from six Chinese public hospitals and their spouses via online questionnaires between April and July 2023. To mitigate common method bias, we implemented a two-wave data collection approach with a one-month interval. We employed confirmatory factor analysis, Pearson’s correlation, and multiple regression to test the hypothesized relationships. The results support the proposed model, indicating that benevolent sexism within intimate relationships moderates the positive effect of job resources on thriving at work, such that this relationship weakens when benevolent sexism within intimate relationships is high. Furthermore, this moderating effect is mediated by self-efficacy, as benevolent sexism within intimate relationships undermines female employees’ self-efficacy, thereby limiting their ability to leverage job resources effectively. These findings highlight the need for human resource managers to consider the personal circumstances of female employees and implement targeted interventions to facilitate their optimal utilization of job resources, thereby enhancing their ability to thrive in both professional and domestic domains.

## 1. Introduction

With the development of the economy and societal progress, women constitute a significant proportion of the workforce, reflecting their growing participation and influence across various sectors. Women’s participation in the labor market has shown an upward trend in recent years. According to the International Labor Organization report, 45.6% of women are estimated to be employed in the global workforce by 2024 (International Labour Organization, 2024). However, gender equality, particularly in the workplace, is still a global issue that needs to be addressed. The United Nations’ sustainable development goals (SDGs) report pointed out that the progress towards gender equality is clearly off track. Due to traditional gender stereotypes, most female employees often have to fulfill their work and family responsibilities simultaneously. Work and family experiences are inherently interconnected, with spillover effects occurring between the two domains (Heller & Watson, 2005). The work–home resources model (Ten Brummelhuis & Bakker, 2012) explains this dynamic by proposing that demands in one domain can deplete personal resources like time and emotional energy, thereby impairing functioning in the other domain. This explains how family demands—whether caregiving responsibilities or interpersonal tensions—ultimately influence employees’ emotional states and effectiveness at work through systematic resource depletion or gain processes.

Studies on female employees have indicated that women often exhibit less competitive behavior in the workplace and are more likely to voluntarily avoid challenging positions that offer higher financial rewards and personal power (Boudreau & Kaushik, 2022; Hanek & Garcia, 2023; Rudman & Heppen, 2003), such as executive managerial roles. This phenomenon is primarily associated with sexism (Dumont et al., 2010; Vescio et al., 2005). Glick and Fiske (1996) introduced the Ambivalent Sexism Inventory (ASI) to capture the dual nature of sexism, distinguishing its two interrelated components: hostile sexism, which overtly devalues women, and benevolent sexism, which, while seemingly portraying women positively, reinforces traditional gender roles (Glick et al., 1997). Benevolent sexism stemming from the family domain may lead women to avoid challenging job opportunities and, in some cases, even give up their careers (Moya et al., 2007). Moreover, women who experience benevolent sexism, particularly from their partners, are more likely to make career trade-offs to prioritize maintaining family relations (Moya et al., 2007). The work–home resources model demonstrates how competing demands between work and family domains deplete psychological resources, negatively impacting functioning across domains (Ten Brummelhuis & Bakker, 2012). Within this framework, benevolent sexism from a partner can be viewed as a family demand that reinforces traditional gender norms and influences how women allocate their limited psychological and physiological resources. This theoretical perspective highlights the critical need to investigate benevolent sexism within intimate relationships as a significant resource drain in work–family dynamics.

Working performance is a common metric to represent the work domain in family–work studies (Carlson et al., 2018). Departing from conventional approaches that focus solely on work performance, this study aims to explore how job resources influence female employees’ ability to thrive at work under the impact of benevolent sexism within intimate relationships. Thriving at work is a positive psychological state in which individuals experience both a sense of vitality and a sense of learning within their working context (Spreitzer et al., 2005). Previous studies have demonstrated that thriving at work enhances task performance (Spreitzer et al., 2012), job satisfaction (Chang & Busser, 2020), and employees’ well-being (Spreitzer et al., 2012). Research shows that female employees’ levels of expertise, professional skills, and communication abilities are closely linked to organizational performance and workplace efficiency (Umar, 2021). Moreover, when female employees are engaged and maintain a positive psychological state, they are more inclined to exhibit higher job performance, consequently improving team dynamics and organizational outcomes (Bai et al., 2024). Promoting positive psychological states among female employees can enhance their well-being and job performance, leading to improved workplace productivity and employee satisfaction. Therefore, understanding female employees’ thriving at work is essential for organizational development. The antecedents of this psychological state include personal traits (Alikaj et al., 2021; Spreitzer et al., 2012), job characteristics (Liu & Bern-Klug, 2013), and organizational context (Kleine et al., 2019). Job resources encompass different aspects of job characteristics that enable individuals to achieve goals and pursue self-learning and development at work (Bakker & Demerouti, 2008; Schaufeli & Bakker, 2004). Moreover, job resources play a pivotal role in generating positive organizational outcomes (Bakker et al., 2014). When employees are able to perceive their jobs as meaningful, feel a sense of personal responsibility, and have a desire to acquire new skills and knowledge, they are more likely to perform actively and focus on personal growth and development within their work context (Hackman, 1980).

This study draws upon the work–home resources model to explain this phenomenon. According to this model, when individuals juggle multiple roles in different domains, the demands from one domain can deplete their personal resources, including affective and cognitive resources (Du et al., 2018). This depletion process leads to an unfavorable outcome where the remaining resources cannot be effectively utilized in the other domain. For example, female employees who experience benevolent sexism from their partners may be required to allocate more attention and time to address family issues, diminishing their energy and enthusiasm for work-related tasks. Among all the personal resources that can impact work performance, self-efficacy plays a crucial role (Stajkovic & Luthans, 1998), especially in situations involving role strain and conflicting demands (Chan et al., 2016). Self-efficacy pertains to one’s confidence in their ability to perform tasks or take action (Bandura, 1977). In the context of work–home resource depletion, self-efficacy becomes critical since individuals with high self-efficacy are better equipped to manage competing demands and overcome challenges in both their work and home domains (Chan et al., 2017). Low levels of self-efficacy can result in reduced work performance, thereby affecting employees’ emotional well-being and job satisfaction (Lee & Ko, 2010). In summary, this study extends the concept of self-efficacy to the work–home context to explain how benevolent sexism within intimate relationships can deplete personal resources in the work domain.

## 2. Literature Review and Hypotheses Materials

### 2.1. Benevolent Sexism Within Intimate Relationships

Benevolent sexism refers to a set of attitudes toward women that appear protective, supportive, and adoring, yet ultimately reinforce traditional gender roles and limit women’s autonomy (Glick & Fiske, 1996). It suggests that women should be cherished and cared for, but simultaneously confines them to stereotypical roles, portraying them as more suited to relational rather than task-oriented domains (Hideg & Shen, 2019). This paradoxical dynamic manifests in everyday interactions—for example, when a female employee withdraws from managerial consideration after receiving ostensibly caring advice like, “Being a manager would be too stressful for you; you’d be happier focusing on family”. Studies demonstrate that women exposed to benevolent sexism within the workplace experience multiple detrimental effects that collectively contribute to impaired job performance (Dardenne et al., 2007; Dumont et al., 2010). The work–home resources model (Ten Brummelhuis & Bakker, 2012) elucidates how benevolent sexist expectations compel women’s disproportionate resource allocation to family domains, thereby depleting their capacity to mobilize work-related resources. These impediments ultimately undermine female employees’ thriving at work—a positive psychological state characterized by simultaneous experiences of vitality (energy and enthusiasm) and learning (continuous development) (Kleine et al., 2019; Spreitzer et al., 2005). Thriving at work represents an active growth process where employees effectively mobilize and transform job resources to both energize work engagement and develop new competencies (Porath et al., 2012). However, as our model demonstrates, benevolent sexism disrupts this dual process by eroding the personal resource required to transform job resources into thriving at work.

This compromised capacity becomes especially critical when examining the essential job resources that facilitate career advancement, including the five core dimensions of skill variety, task identity, task significance, autonomy, and feedback (Hackman, 1980). While the provision of these work-related resources is undeniably important, their ultimate effectiveness depends on how employees perceive and utilize the available opportunities (Beal et al., 2005). Under conditions of benevolent sexism, women tend to direct their efforts toward home and family rather than individual or professional pursuits (King et al., 2012; Oswald et al., 2019), which leaves insufficient resources (e.g., physical energy, time, emotional capacity) to fully engage with workplace opportunities. When personal resources are depleted by competing demands, women may struggle to devote adequate attention and effort to utilize available job resources effectively, even when such resources are formally accessible in the work environment (Du et al., 2018). Moreover, negative emotions arising from family responsibilities can undermine women’s motivation to actively pursue career advancement, thereby constraining their ability to effectively utilize available job resources (Bakker et al., 2014). When individuals face adverse personal circumstances, their capacity to leverage job resources may be substantially weakened. For example, while task significance normally motivates employees by providing purpose, those facing personal struggles may recognize their work’s importance but feel unable to meet expectations. This leads to self-doubt, making them view meaningful tasks as burdens rather than rewards, thereby increasing stress. In addition, researchers suggest that benevolent sexism creates implicit pressures for women to align with conventional gender expectations (Leicht et al., 2017; Rollero & Fedi, 2014). By exclusively valuing women who adhere to traditional roles (Glick & Fiske, 1996), such attitudes may prompt women to downplay their competencies and accomplishments to maintain social acceptance (Horner, 1972; Kosakowska-Berezecka et al., 2017). This represents a trade-off between relationship preservation and professional growth. Overall, the extent to which women translate organizational resources into professional thriving is contingent on benevolent sexism within intimate relationships—which operates by depleting personal resources through imposed familial burdens, changing cognitive approach via emotional expenditure, and displacing career investment with relational priorities. Therefore, the following hypothesis is proposed:

**Hypothesis 1.**  *Benevolent sexism within intimate relationships moderates the relationship between job resources and thriving at work, such that this relationship is weaker when employees experience high (vs. low) levels of benevolent sexism within intimate relationships.*

### 2.2. The Mediating Role of Self-Efficacy

Benevolent sexism perpetuates traditional gender roles that contribute to diminished self-perception and restricted personal agency among women (Glick & Fiske, 1996). Importantly, this dynamic extends to professional domains, where studies demonstrate that exposure to benevolent sexism correlates with reduced self-efficacy, manifesting as lower confidence when confronting challenges (Dardenne et al., 2007; Moya et al., 2007). Self-efficacy refers to an individual’s belief in their ability to successfully perform specific tasks and achieve goals, which significantly impacts how they approach challenges and exert effort in different contexts (Bandura, 1977). In professional settings, a strong sense of self-efficacy is essential for tackling complex challenges and demonstrating competence (Bandura & Locke, 2003), whereas low self-efficacy fosters self-doubt and leads individuals to avoid challenging tasks (Stajkovic & Luthans, 1998).

Benevolent sexism undermines women’s self-efficacy by reinforcing the perception that they are less competent and in need of protection (Connor et al., 2016). When women repeatedly receive unsolicited help and protection, particularly in professional settings, these behaviors act as implicit feedback suggesting they lack the ability to accomplish tasks independently (King et al., 2012). Over time, this can lead to internalized doubts about competence, ultimately weakening their sense of efficacy (Jones et al., 2014). Moreover, the undermining effects extend to social reinforcement of traditional roles. In environments where benevolent sexism persists, women may internalize these beliefs (Rollero & Fedi, 2014), becoming less confident in their skills and more hesitant to take on challenges (Leicht et al., 2017). This system rewards conformity to traditional gender roles with social approval, while deviation risks disapproval or exclusion (Kosakowska-Berezecka et al., 2017). Consequently, women, especially those with benevolently sexist partners, may avoid competitive behaviors or downplay ambitions to preserve relationship harmony (Horner, 1972), further eroding self-efficacy.

Therefore, the following hypothesis is proposed:

**Hypothesis 2.**  *Benevolent sexism within intimate relationships is negatively related to self-efficacy.*

Self-efficacy is a key personal resource that enables individuals to effectively utilize job resources, which in turn is associated with positive job performance (Bakker & Demerouti, 2008). By influencing the extent to which employees perceive the available job resources, self-efficacy may also determine their feelings toward well-being (Loeb et al., 2016). Since thriving at work is influenced by both individual and external factors, employees’ ability to effectively leverage job resources plays a crucial role in fostering their thriving (Walumbwa et al., 2020). As a personal resource, self-efficacy determines how employees perceive and utilize these job resources (Xanthopoulou et al., 2007), thereby shaping their ability to thrive in the workplace.

Employees with high self-efficacy are more likely to perceive job resources as tools for success and actively utilize them to enhance performance and professional growth (Luthans, 2002; Xanthopoulou et al., 2007). High self-efficacy also enables employees to remain engaged and persistent when encountering challenges, allowing them to make full use of job resources to support their development (Mazzetti et al., 2023). As a result, job resources become catalysts for thriving at work rather than mere external conditions.

In contrast, employees with lower levels of self-efficacy cannot obtain the energy to remain committed to completing their job tasks (De Clercq et al., 2018). Furthermore, a low sense of self-efficacy always leads to negative affect, such as depression, anxiety, and helplessness (Muris, 2002; Schwarzer et al., 1997). Due to limited confidence in their abilities, these employees may struggle to identify and utilize job resources effectively, reducing the likelihood that such resources will contribute to thriving. In addition, inadequate self-efficacy leads to low self-esteem and generates skeptical thoughts toward achievements and career development (Schwarzer et al., 1997). Consequently, employees with low self-efficacy may fail to translate available job resources into opportunities for growth, ultimately hindering thriving at work. Therefore, the following hypothesis is proposed:

**Hypothesis 3.**  *Self-efficacy moderates the relationship between job resources and thriving at work, such that this relationship is stronger for employees with high (vs. low) levels of self-efficacy.*

Based on Hypotheses 1 to 3, benevolent sexism within intimate relationships negatively affects women employees’ self-efficacy. This reduced self-efficacy is associated with decreased ability to effectively utilize job resources, ultimately leading to lower levels of thriving at work. The work–home resources model suggests that demands from one life domain can negatively impact another domain by depleting psychological resources (Ten Brummelhuis & Bakker, 2012). Thus, the effect of benevolent sexism within intimate relationships is moderated through its influence on self-efficacy. Research provides empirical support for self-efficacy’s mediating role between contributing factors and strain across work and family domains (Chan et al., 2020). These findings collectively support the following hypothesis:

**Hypothesis 4.**  *Self-efficacy mediates the moderating effect of benevolent sexism within intimate relationships on the relationship between job resources and thriving at work.*

**Figure 1:**
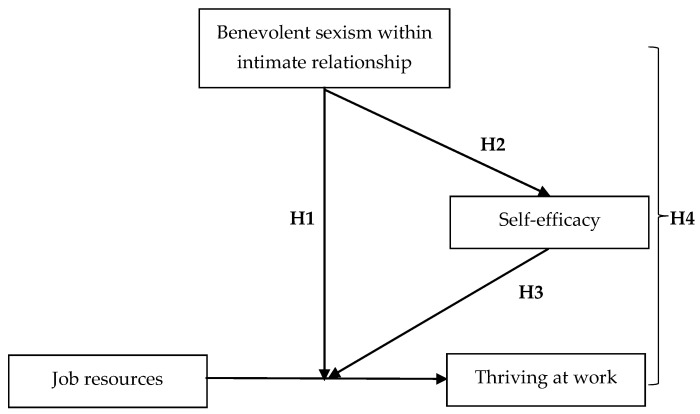
Proposed mediated moderation model.

To sum up, in line with the notion that family-to-work interference occurs when demands from the family domain reduce personal resources, compromising the efficiency of job resources utilization and ultimately producing negative results in the work domain (Ten Brummelhuis & Bakker, 2012), this study proposes that benevolent sexism within intimate relationships reduces female employees’ self-efficacy, ultimately impeding the utilization of job resources and hindering thriving at work. Therefore, this study proposes the following research framework (see Figure 1).

## 3. Research Methods

### 3.1. Sampling and Data Collection

Participants for the surveys were recruited through snowball sampling, which is particularly suitable for the Chinese context (Sun et al., 2007) due to the importance of social networks in facilitating trust and participation in research. A total of 350 sets of questionnaires were distributed. The participants included married female nurses from six public hospitals located in four cities and regions in China: Guangzhou, Guiyang, Nanchang, and Nanning, along with their husbands.

The questionnaires were administered electronically, with separate survey links provided for female nurses and their spouses to ensure independent responses. The husbands were contacted through the female nurses, who forwarded the electronic survey links to their spouses after obtaining their consent. To ensure accurate matching of questionnaires between female nurses and their spouses, we included a unique identifier in both surveys. Female nurses were asked to provide the last four digits of their birth date (e.g., January 1 as ‘0101’), and their spouses were asked to provide the last four digits of their wife’s birth date. This allowed us to match the responses from each nurse–spouse pair for subsequent analysis. Female nurses were selected as the sample because they represent a significant portion of the female workforce in healthcare, a sector known for its high demands and unique work–family balance challenges. According to the China Health Statistics Yearbook (National Health Commission of the People’s Republic of China, 2023), by the year 2022, female nurses accounted for 96.5% of the total number of registered nurses in China. This demographic dominance makes female nurses a representative group for examining the effects of partner benevolent sexism on self-efficacy and job-related outcomes. To mitigate common method bias, we employed a two-stage data collection process with a one-month interval between the stages. During the initial survey phase (Time 1), facilitated by hospital managers, we distributed 350 sets of questionnaires to married female nurses and their spouses. The questionnaire header included detailed information about the study’s purpose and procedures, and emphasized informed consent, data anonymization, and confidentiality. The female nurses completed surveys that assessed demographic information and job resources, while their spouses completed surveys that evaluated demographic information and benevolent sexism within intimate relationships. By the conclusion of the first stage, we received completed questionnaires from 293 female nurses, resulting in a response rate of 83.71%, and from 268 of their spouses, resulting in a response rate of 76.57%. The higher response rate among female nurses may be attributed to the facilitation by hospital managers, whereas their husbands, being external to the hospital environment, may have been less motivated or accessible. One month later (Time 2), we conducted the second wave of the survey, which concentrated on measuring self-efficacy and thriving at work. This second phase yielded 281 completed questionnaires, corresponding to a response rate of 80.29%. After completing this phase, we performed questionnaire matching and successfully obtained 209 valid matched sets, resulting in an overall response rate of 59.71%. Of these female nurses, 72.2% had completed at least a college education. The average age of the participants was 32.5 years (SD = 6.7), with an average work tenure of 9.0 years (SD = 6.5) and an average of 0.98 children (SD = 0.84). In the matching sample of male spouses, the majority are aged between 31 and 40 (52.3%), and the majority have an undergraduate education (56.4%).

### 3.2. Measures

The questionnaires, initially developed in English, were administered in Chinese following a rigorous translation and back-translation process to ensure linguistic and conceptual equivalence between the two versions. Specifically, this study employed the standard translation and back-translation procedures recommended by Brislin (Brislin, 1970). The scales were first translated from English to Chinese and then back-translated to English by two professors specializing in organizational behavior. After three rounds of forward–backward–forward translation, the content of the scales achieved consistency in both semantics and wording. Except for demographic information, all variables were measured using 5-point Likert-type scales, ranging from 1 (strongly disagree) to 5 (strongly agree).

The variable job resources was measured with a 15-item scale (Hackman & Oldham, 1975), which encompasses five dimensions: autonomy, feedback, skill variety, task identity, and task significance. Examples of items for each dimension include: “I decide on my own how to go about doing the work” (autonomy); “When I finish a job, I know whether I performed well” (feedback); “The job requires me to perform a variety of tasks” (skill variety); “The job provides me the chance to completely finish the piece of work I begin” (task identity); and “The job itself is very significant and important in the broader scheme of things” (task significance). The Cronbach’s α for this scale was 0.90.

Benevolent sexism within intimate relationships (BSIR) was measured using an 11-item sub-scale of benevolent sexism from the Ambivalent Sexism Inventory (ASI) (Glick & Fiske, 1996). A sample item is “A good woman should be set on a pedestal by her man”. The Cronbach’s α for this scale was 0.79.

Self-efficacy was measured using a 10-item scale (Schwarzer et al., 1997). A sample item is “I can always manage to solve difficult problems if I try hard enough”. The Cronbach’s α for this scale was 0.92.

Thriving at work (TAW) was measured using a 10-item scale (Porath et al., 2012), which includes two dimensions: vitality and learning. Sample items include “I feel alive and vital (vitality)” and “I find myself often learning (learning)”. The Cronbach’s α for this scale was 0.87.

Control variables: In addition to job resources, job demands represent another key category within the work environment (Bakker et al., 2014). Considering the importance of job demands in influencing employees’ psychological state and job performance (Bakker & Demerouti, 2017), this study controls for job demands to isolate the specific effects of contextual demands from the family domain. We measured job demands with Job Content Instrument (Karasek et al., 1998). An example item is “My job requires working very fast”. Furthermore, research on thriving at work has suggested that demographic variables can exert certain effects (Abid et al., 2020). Particularly, other control variables in our study include age and tenure.

## 4. Results

### 4.1. Measurement Model

We conducted a series of confirmatory factor analyses (CFA) to examine the discriminant validity of all research variables (see Table 1). As depicted in Table 1, the hypothesized four-factor model, which includes job resources, benevolent sexism within intimate relationships, self-efficacy, and thriving at work, demonstrated a significantly superior fit [χ^2^/983 = 1.35; comparative fit index (CFI) = 0.91; Tucker-Lewis Index (TLI) = 0.91; root mean square error of approximation (RMSEA) = 0.06; standardized root mean residual (SRMR) = 0.04] compared to alternative models, including three-factor, two-factor, and one-factor models. Additionally, the chi-square difference test indicated that the hypothesized model provided the best fit to the data, thereby supporting the discriminant validity of the measures.

### 4.2. Descriptive Statistics and Correlations

The descriptive statistics and bivariate correlations among the study variables are presented in Table 2. The findings revealed a positive correlation between job resources and thriving at work (r = 0.26, *p* < 0.01). Additionally, benevolent sexism within intimate relationships displayed a negative association with self-efficacy (r = −0.23, *p* < 0.01). These results provide initial support for the proposed hypotheses.

### 4.3. Hypothesis Testing

Building on our dyadic research design, Hypothesis 1 proposed that benevolent sexism within intimate relationships moderates the relationship between job resources and thriving at work. Notably, the benevolent sexism measures were obtained from husbands’ reports, providing an objective assessment of relationship dynamics. As shown in Table 3, the interaction term was significant (*b* = −0.33, *p* < 0.01). Simple slope tests revealed that when husbands reported low levels of benevolent sexism (1 SD below the mean), job resources showed a strong positive association with thriving (*b* = 0.38, *p* < 0.001). However, when husbands reported high benevolent sexism (1 SD above the mean), this beneficial relationship became nonsignificant (*b* = −0.09, *p* > 0.05), supporting Hypothesis 1.

Hypothesis 2 examined the cross-partner effect between benevolent sexism within intimate relationships and self-efficacy. Consistent with the dyadic nature of our data, results showed that husbands’ reports of benevolent sexism significantly predicted their wives’ lower self-efficacy (*b* = −0.33, *p* < 0.001). This finding underscores how partners’ attitudes can shape women’s professional self-perceptions, providing strong support for Hypothesis 2.

For Hypothesis 3, we tested whether self-efficacy moderates the job resources-thriving relationship. The significant interaction (*b* = 0.30, *p* < 0.01) indicated differential effects: job resources enhanced thriving when self-efficacy was high (*b* = 0.45, *p* < 0.01), but showed no significant effect when self-efficacy was low (*b* = −0.07, *p* > 0.05). This pattern held regardless of husbands’ benevolent sexism levels, confirming the robustness of self-efficacy’s moderating role.

**Figure 2:**
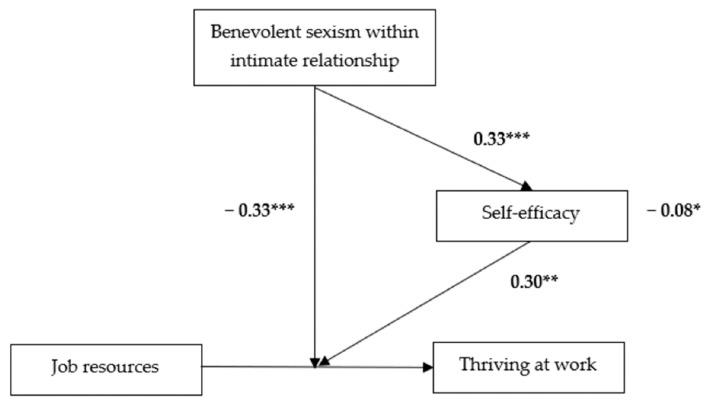
Empirical Results of the Mediated Moderation Model. Note: Unstandardized coefficients (*b*) are shown (* *p* < 0.05, ** *p* < 0.01, *** *p* < 0.001). The indirect effect was significant (*b* = −0.084, 95% CI [−0.181, −0.034]. The job resources → thriving relationship was nonsignificant at high BSIR (*b* = −0.09).

**Figure 3:**
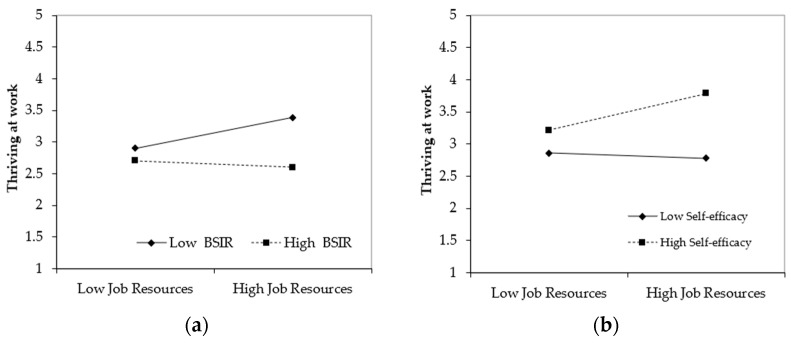
The moderating effect. Note: (**a**) Moderating effect of benevolent sexism within intimate relationships on job resources–thriving at work relationship. (**b**) Moderating effect of self-efficacy on job resources–thriving at work relationship.

The mediated moderation analysis supported Hypothesis 4 (indirect effect = −0.084, 95% CI [−0.181, −0.034]). These results reveal an important interpersonal dynamic: when husbands endorse benevolent sexism, it undermines their wives’ self-efficacy, which in turn diminishes the positive impact on job resources on thriving at work (See Figure 2 and Figure 3). This pattern aligns with our theoretical model of cross-domain resource depletion, where relationship dynamics shape work-related outcomes through psychological mechanisms.

## 5. Discussion

This study reveals that husband-reported benevolent sexism within intimate relationships undermines female employees’ work thriving by reducing their self-efficacy, thereby weakening the positive impact on job resources. The findings demonstrate this cognitive spillover process from family to work domains, particularly when benevolent sexism levels are high, offering new insights into how traditional gender roles shape work outcomes through interpersonal dynamics. We now elaborate on the theoretical and practical significance of these findings.

### 5.1. Theoretical Contributions

This study contributes to existing literature in the following three aspects. First, it provides valuable insights into the family–work dynamic by demonstrating how benevolent sexism within intimate relationships moderates the relationship between job resources and thriving at work. Unlike previous research that relied on participants’ self-reports of work–family interference—a method potentially biased by personal status and social desirability—our study adopted a dyadic approach by collecting data from both female nurses and their husbands. This design minimizes subjective bias and offers a more nuanced understanding of how family dynamics influence work outcomes. By capturing the dyadic nature of family–work interactions, our study reveals how benevolent sexism within intimate relationships operates as an internal family demand that spills over into the work domain, bridging the gap between family and work research and offering a more comprehensive framework for understanding the interplay between these two domains.

Second, our study reveals the mechanism of cognitive spillover by demonstrating that self-efficacy mediates the moderating effect of benevolent sexism within intimate relationships. While research on the spillover process has mainly focused on affective spillover between domains, few studies have explored cognitive spillover from the family to the work domain (Offer, 2014). Our findings suggest that stressors from the family domain can lead to a depletion of cognitive resources in the work domain, reducing the potential to utilize job resources. This aligns with the perspective of the work–home resources model (Ten Brummelhuis & Bakker, 2012), which posits that the family domain influences the work domain by consuming personal resources. By incorporating data from husbands, we were able to capture the dyadic nature of family–work interactions, demonstrating how a partner’s attitudes directly impact female employees’ cognitive resources and work outcomes.

Third, this study enriches empirical studies on benevolent sexism by shifting the focus from its general measurement to its manifestation within intimate relationships. While previous research has predominantly examined benevolent sexism as a phenomenon measured in general contexts (Cheng et al., 2019; Hideg & Ferris, 2016), our study demonstrates its significant impact from the family domain on female employees’ work outcomes. Specifically, we identify a clear mechanism through which benevolent sexism within intimate relationships hinders female employees’ utilization of job resources, ultimately reducing their ability to thrive at work. This mechanism, mediated by self-efficacy, highlights how cognitive resources are depleted when female employees experience benevolent sexism from their spouses.

### 5.2. Practical Implications

This study offers three implications for contemporary human resources management, particularly in the Chinese context, where Confucian values emphasize familial harmony and women’s traditional roles as caregivers, while recognizing the government’s commitment to gender equality under socialist core values. These recommendations specifically address how organizations can counteract benevolent sexism’s unique impacts.

First, benevolent sexism within intimate relationships hinders female employees’ task-related performance, reflecting the tension between traditional gender norms and modern workplace demands in Chinese society. Organizations should implement targeted interventions that combine Confucian family values with state-sponsored gender equality initiatives, such as offering training programs for spouses through collaboration with local women’s federations that highlight research on benevolent sexism’s workplace consequences, while providing female employees with workshops that incorporate successful Chinese female role models from various professions who have navigated similar gender biases.

Second, benevolent sexism within intimate relationships impairs the full potential of job resources, diminishing thriving at work due to deficient self-efficacy. Therefore, organizations should develop programs that draw upon China’s collectivist culture, such as group-based mentorship initiatives pairing female employees with senior leaders who exemplify both professional achievement and family virtue, and training sessions that use culturally resonant examples of women overcoming gender barriers in Chinese workplaces with particular attention to subtle benevolent sexism manifestations.

Third, organizational leaders should adapt China’s family-friendly policy framework to create supportive environments. This could involve establishing workplace–family liaison programs that mediate between employees and their families, organizing traditional festival-based family events to foster mutual understanding while challenging benevolent sexism stereotypes, and developing resources that help employees navigate work–family conflicts within the context of China’s unique social support systems through evidence-based approaches to gender equity.

### 5.3. Limitations and Future Directions

There are several limitations in this study. First, as a cross-sectional study, despite the one-month interval between data collections, this study cannot establish causality with certainty. Future research could adopt experimental or multi-wave longitudinal designs to strengthen causal inferences. Moreover, while this study utilized a dyadic data collection approach by incorporating responses from both female employees and their husbands, the analytical integration of these responses could be further refined. Future research could explore more sophisticated dyadic analysis techniques to better capture the interactive dynamics between partners.

Second, this study was conducted with a limited number of nurses from a few regions, restricting both the geographical scope and sample size. Furthermore, as the sample consisted solely of Chinese nurses, the generalizability of our findings to other cultural contexts or occupational groups remains uncertain. Future research should examine whether similar patterns hold in different professional and sociocultural settings. In addition, while this study primarily focuses on the effect of benevolent sexism within intimate relationships on female nurses at the individual level, future research could delve into key organizational and social elements, such as organizational justice and social equality.

Third, our findings indicate that the relationship between job resources and thriving at work was significant only when benevolent sexism within intimate relationships was low, whereas this relationship became statistically nonsignificant when benevolent sexism within intimate relationships was high. However, we observed a declining trend in thriving at work when benevolent sexism within intimate relationships was high, regardless of job resource levels. This suggests that although the current sample size may have limited the statistical power to detect significant effects, an expanded sample in future studies could provide a more robust examination of this interaction.

Fourth, while the original Ambivalent Sexism Inventory (ASI) was validated using a six-point Likert scale without a neutral midpoint (Glick & Fiske, 1996), our study adapted it to a 5-point scale to maintain consistency with other measures in our survey battery. This modification may have influenced response patterns, as evidenced by the higher mean score (M = 3.66, SD = 0.70) compared to norms established with the six-point scale. Future research should maintain the original scaling to ensure direct comparability with established benchmarks.

Fifth, our study focused primarily on the impact of a partner’s benevolent sexism on women’s job performance without accounting for the potential internalization of these beliefs by the women themselves. While research (e.g., Moya et al., 2007) suggests that self-endorsement of benevolent sexism may buffer its negative effects, our design did not assess whether participants personally adhered to such beliefs. Future studies could explore the interplay between partner-derived and self-endorsed benevolent sexism to better understand their combined effects on work performance.

Finally, while our findings suggest that benevolent sexism weakens the relationship between job resources and thriving at work, this moderating effect was not statistically significant under high benevolent sexism conditions. This could be due to limited sample size reducing statistical power, or potential measurement constraints (e.g., our adaptation of the ASI scale from six to five points). Future studies with larger and more diverse samples, as well as stricter adherence to the original scale format, could help clarify whether job resources truly become less impactful for thriving when benevolent sexism is high.

## 6. Conclusions

This study demonstrates that benevolent sexism in intimate relationships significantly weakens the positive effect of job resources on female employees’ thriving at work, with self-efficacy serving as the key mediating mechanism. These findings validate the work–home resources model by revealing how family-domain factors can constrain women’s ability to benefit from workplace resources. The results call for organizational interventions that simultaneously address job resource allocation and gender bias in personal relationships to enhance women’s career development. Future research should investigate how these dynamics vary across different industries and cultural settings.

## Figures and Tables

**Table 1:** Confirmatory factor analysis (CFA) results.

Model	χ^2^	df	χ^2^/df	CFI	TLI	SRMR	RMSEA
Four-factor model	1322.95	983	1.35	0.91	0.91	0.06	0.04
Three-factor model	1832.49	986	1.86	0.78	0.77	0.08	0.06
Two-factor model	2781.44	988	2.82	0.53	0.51	0.13	0.09
One-factor model	3110.86	989	3.15	0.44	0.42	0.14	0.10

Note: CFI = Comparative Fit Index. TLI = Tucker-Lewis Index. SRMR = Standardized Root Mean Square Residual. RMSEA, root mean square error of approximation. Three-factor model: combining self-efficacy and thriving at work. Two-factor model: combining job resources, self-efficacy, and thriving at work. One-factor model: combining all research variables.

**Table 2:** Descriptive statistics and correlations.

Variable	M	SD	1	2	3	4	5	6
1. Age	32.51	6.72						
2. Tenure	9.03	6.55	0.72 **					
3. Job demands	3.35	0.87	0.01	−0.03				
4. Job resources	3.95	0.64	0.18 *	0.17 *	0.36 **			
5. BSIR	3.66	0.70	0.05	0.02	0.18 **	0.05		
6. Self-efficacy	3.59	0.85	−0.12	−0.04	0.25 **	0.19 **	−0.23 **	
7. Thriving at work	3.38	0.82	0.02	0.11	0.23 **	0.26 **	−0.28 **	0.50 ***

Note: *N* = 209, Abbreviations: BSIR, benevolent sexism within intimate relationships. * *p* < 0.05, ** p < 0.01, *** *p* < 0.001.

**Table 3:** Regression results for total, direct and indirect effects.

	Thriving at Work										Self-Efficacy	
	MODEL1		MODEL2 X → Y		MODEL3 X, W → Y		MODEL4 X, M → Y		MODEL5 X, W, M → Y		MODEL6 W → M	
**Variable**	Estimate	SE	Estimate	SE	Estimate	SE	Estimate	SE	Estimate	SE	Estimate	SE
**Constant**	2.90 ***		2.23 ***		3.60 ***		0.98 ***					
**Age**	−0.02	0.01	−0.02	0.01	−0.01	0.01	−0.01	0.01	−0.01	0.01	−0.02	0.01
**Tenure**	0.03 *	0.01	0.02	0.01	0.02	0.01	0.02	0.01	0.02	0.01	0.01	0.01
**JD**	0.23 **	0.08	0.16 *	0.08	0.22 **	0.07	0.08	0.07	0.12	0.07	0.29 ***	0.08
**JR**			0.25 ***	0.07	0.15	0.08	0.19 **	0.07	0.14	0.07		
**BSIR**					−0.35 ***	0.07			−0.19 *	0.08		
**JR × BSIR**					−0.33 **	0.11			−0.20	0.11		
**S. Eff**							0.40 ***	0.06	0.34 ***	0.07		
**JR ×** **S.Eff**							0.30 **	0.09	0.25 **	0.08		
**R^2^**	0.08		0.11		0.24		0.34		0.35			
**ΔR^2^**			0.03		0.16		0.26		0.27			
**F**	5.94 **		6.03 **		10.63 **		17.34 **		13.46 **			

Note: *N* = 209. Abbreviations: BSIR, benevolent sexism within intimate relationships. X, job resources. Y, thriving at work. W, benevolent sexism within intimate relationships. M, self-efficacy. JD, job demands. JR, job resources. S. Eff, self-efficacy. SE, Standard error. ΔR^2^, Change in R-squared. * *p* < 0.05, ** *p* < 0.01, *** *p* < 0.001.

## Data Availability

The data shown in this research are available on request from the corresponding author.
